# Supplementary material for: Generation of a novel mouse model of nemaline myopathy due to recurrent NEB exon 55 deletion
Source: Skelet Muscle. 2025 Mar 20;15:8. doi: 10.1186/s13395-025-00378-2 (PMC11924678; doi:10.1186/s13395-025-00378-2)
Supplement: Supplementary file 1 — Supplementary Material 1 [file 13395_2025_378_MOESM1_ESM.docx]

**Supplementary Figures**

**Figure S1.**

*Neb*^ΔExon55^ Pseudoexon Nucleotide Sequence:

TTTGATCCTGAAGGCCTAATTAACTTCTCTGAGCCATTTGCATGATAGAAAGCATATTCCCGCGGTGGCGGCCGCTCTAGAACTAGTGGATCCCCCGGGAAGTTCCTATACTTTCTAGAGAATAGGAACTTCGGAATAGGAACTTCGTCGATCGACCTCGAATCGATACCGTCGACAACAACAGTAGACAATGGATTTCTGG

*Neb*^ΔExon55^ Pseudoexon Amino Acid Sequence:

FDPEGLINFSEPFA**KAYSRGGGRSRTSGSPGKFLYFLENRNFGIGTSSIDLESIPSTTTVDNGFL

HMZ- *Neb*^ΔExon55^ Humanized Allele Sequence:

CTGGCGTTCCTACCAGAAAGTTAGCAGGTAGAGAAACTCTGCTGTTTGCTTGGTGGGTACAATGTGTGTTGCTCCAGTCAGAGGACAGAGTTCCTTTC

**Figure S1 DNA and amino acid pseudoexon sequence from RNAseq and humanized allele sequence.** The pseudoexon includes 59bp of *Neb* intron 54 sequence (black) followed by 117bp of the residual vector sequence from the methods previously used to establish this model (red), followed by 26bp of intron 55 (black). For the amino acid sequence stars (*) are a place holder for stop sequence. The pseudoexon forming in *Neb*^ΔExon55^ mice contains two stop codons. The humanized allele sequence includes 20bp of mouse *Neb* intron 54 followed by the 58bp human *NEB* intron 54 sequence highlighted in red followed by 20bp of mouse intron 55.

**Figure S2.**


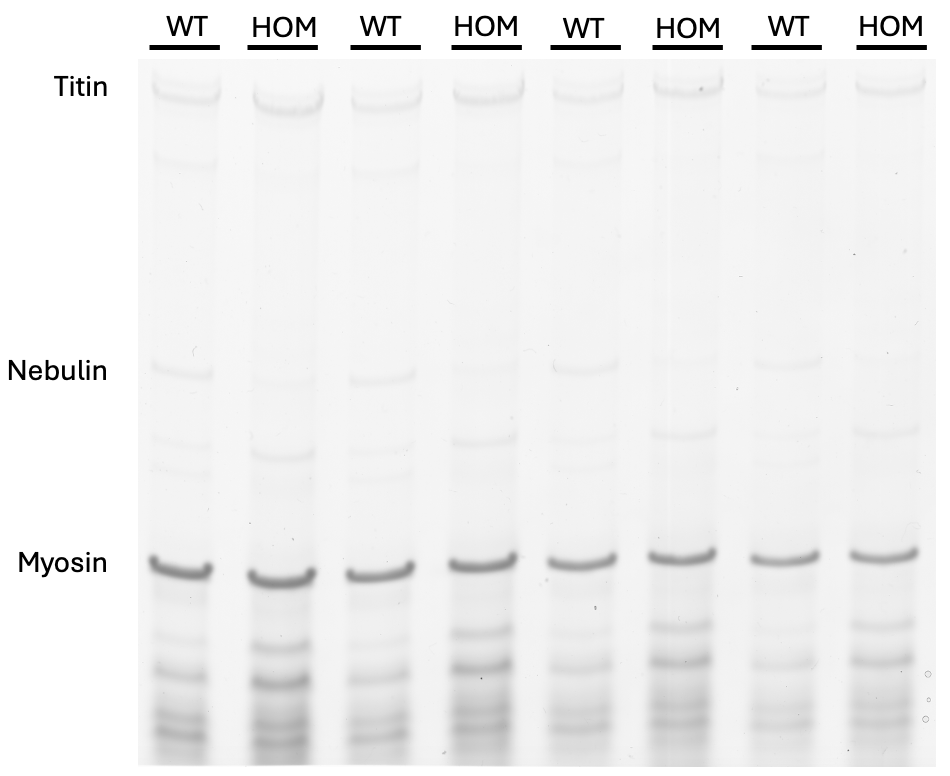


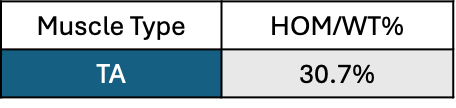


**Figure S2 Hmz-*Neb*^ΔExon55^** **protein expression analysis by analysis gel.** Protein gel of WT and HOM male Hmz-*Neb*^ΔExon55^ TA muscle. Nebulin expression levels are reduced to 30.7% WT expression levels when normalized to titin.

**Figure S3.**

**Figure S3** ***Neb*^ΔExon55^ animal survival.** Kaplan-Meier survival curve comparing *Neb*^ΔExon55^ WT and HOM survival. HOM animals have a median survival of less than 1 day and a mean survival of 4 days of age.

**Figure S4.**

0.0071

<0.0001

<0.0001

<0.0001

0.0453

**Figure S4 Muscle mechanics tissue normalizers.** Normalizers used for EDL and SOL muscle mechanics analysis in ~3-month-old Hmz- *Neb*^ΔExon55^ mice. HOM animals have a significant (P<0.0001) reduction in EDL physiological cross-sectional area (PCSA), female animals have a significant (P=0.0071) increase in SOL PCSA, and male and female HOM animals have a significant (P<0.0001, P=0.0453) reduction in body weight. Tibia length is unchanged between WT and HOM animals.

**Figure S5.**


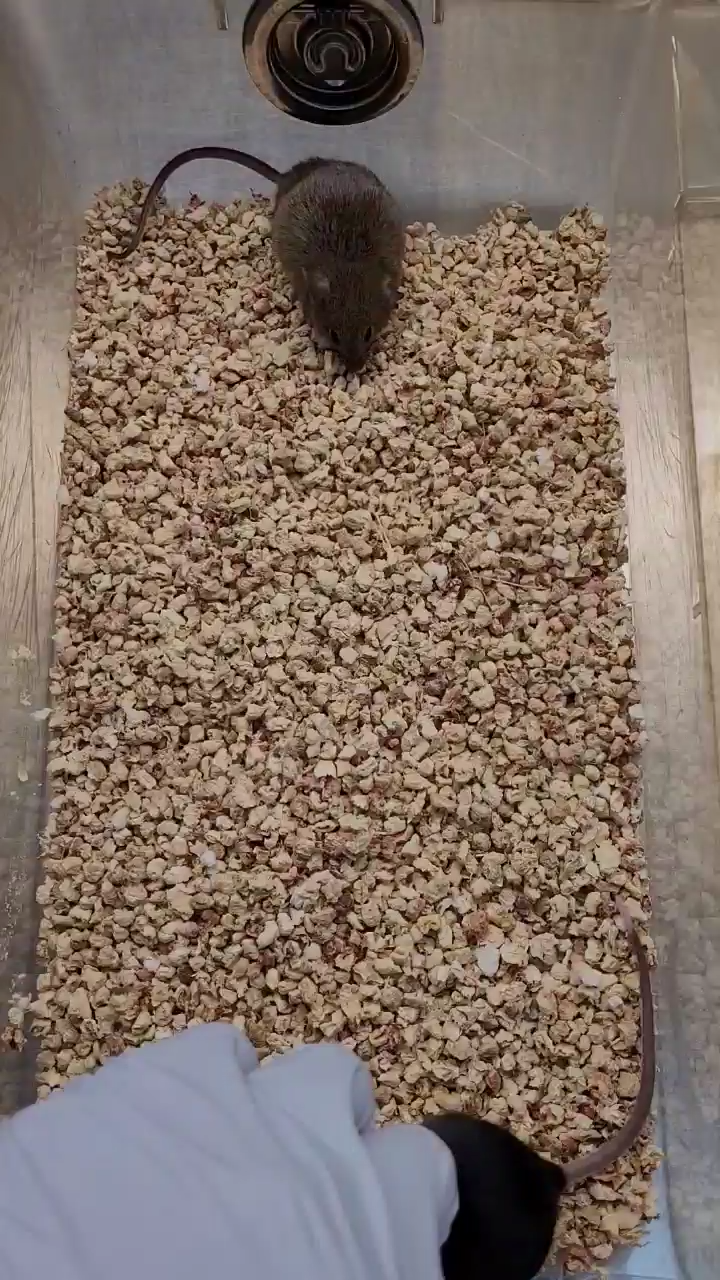


**Figure S5 Hmz-*Neb*^ΔExon55^** **visual motor function.** Video comparing ~2.5-month-old Hmz-*Neb*^ΔExon55^ WT (black) and HOM (brown) mice. The HOM animal appears smaller and less responsive than its WT littermate with slower and more laboured movement indicative of nemaline myopathy.

**Figure S6.**


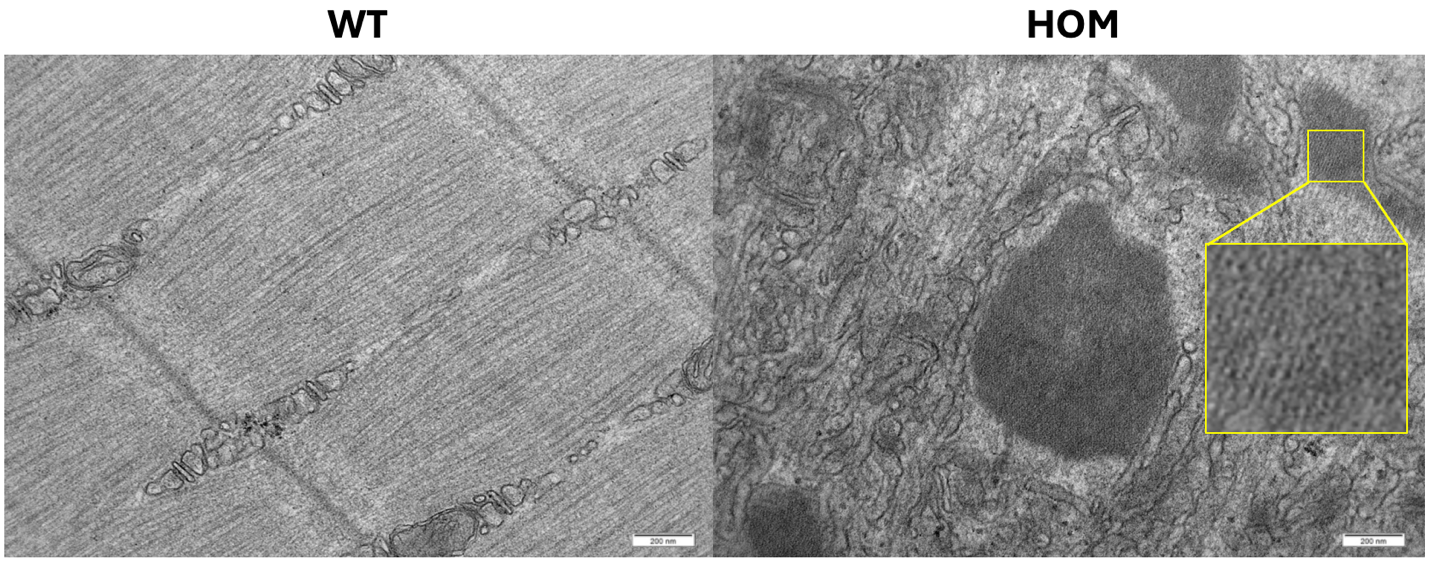


**Figure S6 Nemaline rod structure.** TEM imaging of glutaraldehyde fixed longitudinal TA sections from *n*=4 ~2-month-old WT and HOM male Hmz-*Neb*^ΔExon55^ mice demonstrating the filamentous structure observed in many of the nemaline rods.

**Figure S7.**

1- *Neb*^ΔExon55 +/+^

Exon 54

Exon 55

Exon 56

4- Hmz*-Neb*^ΔExon55 +/+^

3- Hmz*-Neb*^ΔExon55 +/+^

2- Hmz*-Neb*^ΔExon55 +/+^

1- Hmz*-Neb*^ΔExon55 +/+^

2- *Neb*^ΔExon55 +/+^

**Figure S7 WT control RNAseq reads for *Neb*^ΔExon55^ and Hmz*-Neb* ^ΔExon55^ mice.** Sashimi plots displaying WT RNAseq reads from both *Neb* mouse lines showing proper exon sequence usage and normal splicing.
